# 3′-End Processing of Eukaryotic mRNA: Machinery, Regulation, and Impact on Gene Expression

**DOI:** 10.1146/annurev-biochem-052521-012445

**Published:** 2023-03-31

**Authors:** Vytautė Boreikaitė, Lori A. Passmore

**Affiliations:** Medical Research Council Laboratory of Molecular Biology, Cambridge, United Kingdom

**Keywords:** pre-mRNA processing, endonuclease, polyadenylation, poly(A) tail, polymerase, transcription

## Abstract

Formation of the 3′end of a eukaryotic mRNA is a key step in the production of a mature transcript. This process is mediated by a number of protein factors that cleave the pre-mRNA, add a poly(A) tail, and regulate transcription by protein dephosphorylation. Cleavage and polyadenylation specificity factor (CPSF) in humans, or cleavage and polyadenylation factor (CPF) in yeast, coordinates these enzymatic activities with each other, with RNA recognition, and with transcription. The site of pre-mRNA cleavage can strongly influence the translation, stability, and localization of the mRNA. Hence, cleavage site selection is highly regulated. The length of the poly(A) tail is also controlled to ensure that every transcript has a similar tail when it is exported from the nucleus. In this review, we summarize new mechanistic insights into mRNA 3′-end processing obtained through structural studies and biochemical reconstitution and outline outstanding questions in the field.

## 1 Introduction

The complexity of the eukaryotic cell is enabled by multiple intricate gene-regulatory mechanisms. In the nucleus, these include regulation of transcription as well as extensive cotranscriptional processing of precursor messenger RNAs (pre-mRNAs). Pre-mRNA processing in eukaryotes includes 5′capping with a 7-methylguanosine cap, removal of intronic sequences by the splicing machinery, and 3′-end processing (1). All three steps must be completed for themRNA to be efficiently exported out of the nucleus and translated in the cytoplasm, and thus, pre-mRNA processing is critical to the production of functional protein-coding transcripts. Regulation of pre-mRNA processing increases the diversity of protein products generated from a single gene and modifies how their expression is regulated posttranscriptionally (2). Recent years have witnessed major progress in our understanding of how pre-mRNAs are processed at their 3 ’ends, how this is regulated, and how it contributes to disease (3).

**Figure 1:**
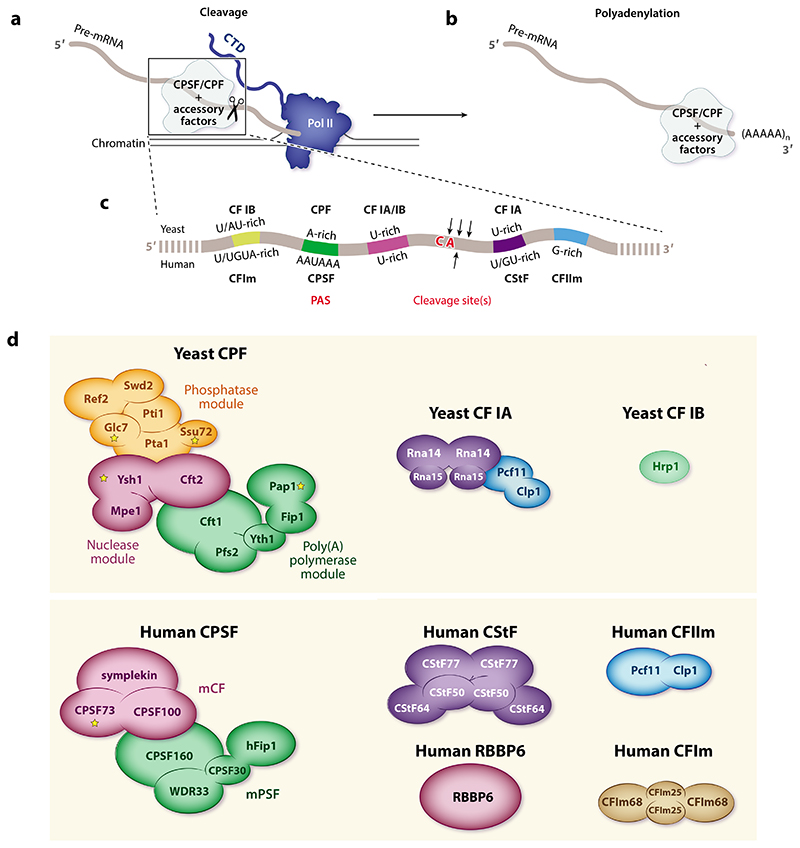
3′-end processing depends on multiple protein complexes and cis-regulatory elements. Schematic representations of cotranscriptional (*a*) cleavage and *(b)* polyadenylation reactions catalyzed by CPSF and regulated by accessory factors. (*c*) The pre-mRNA region carrying the cis-regulatory elements required for 3′-end processing. Protein factors binding to each element in yeast (*top*) and humans (*bottom*) are indicated. *(d)* Schematics of the proteins required for 3′-end processing. Panel *c* adapted from Reference 7; panel *d* adapted from Reference 10. Abbreviations: CF, cleavage factor; CPF, cleavage and polyadenylation factor; CPSF, cleavage and polyadenylation specificity factor; CStF, cleavage stimulatory factor; CTD, C-terminal domain; mCF, mammalian cleavage factor; mPSF, mammalian polyadenylation specificity factor; PAS, polyadenylation signal; Pol II, RNA polymerase II; poly(A), polyadenosine; pre-mRNA, precursor messenger RNA.

3′-end processing of eukaryotic pre-mRNAs includes a cotranscriptional endonucleolytic cleavage event at a specific site in the pre-mRNA (Figure 1a). This releases the nascent transcript from RNA polymerase II (Pol II) and generates a free 3′end that is a substrate for the addition of a polyadenosine [poly(A)] tail, which in turn is required for nuclear export and efficient translation of the mRNA (Figure 1b) (4). The site of cleavage defines the 3′end of the mature transcript and therefore determines the C-terminal sequence of the protein product and/or the length and sequence of the 3′untranslated region (UTR) of the mature mRNA. 3′UTR sequences regulate the translational efficiency, localization, and stability of the mRNA, as well as the localization and activity of its protein product (2). 3′-end processing is also intimately linked to other cotranscriptional processes such as splicing and transcription termination (5).

Most of our understanding of pre-mRNA 3′-end processing stems from studies of human and budding yeast systems, and the general mechanisms are highly conserved across eukaryotes. Over 80 proteins have been identified as part of the eukaryotic 3′-end-processing machinery, either directly involved in cleavage and polyadenylation reactions or coordinating them with other nuclear processes (6).

3‘-end processing in eukaryotes requires many protein factors that recognize cis-regulatory elements surrounding the cleavage site (Figure 1c,d). In particular, cleavage and polyadenylation are carried out by a large multi-subunit protein complex, termed cleavage and polyadenylation specificity factor (CPSF) in humans and cleavage and polyadenylation factor (CPF) in yeast (Figure 1d) (7). The CPSF/CPF complexes host multiple enzymatic subunits, including an endonuclease (CPSF73 in humans, Ysh1 in yeast) and a poly(A) polymerase (PAP in humans, Pap1 in yeast), that catalyze the two steps of pre-mRNA 3′-end processing (8, 9). Two protein phosphatases are also part of CPF but are peripherally associated with CPSF (6, 9). In addition, multiple accessory protein factors have been implicated in the activation and regulation of both cleavage and polyadenylation (Figure 1d). For example, cleavage factors activate the CPSF/CPF endonuclease, and most of these can be considered part of the active 3′-end-processing machinery. These include cleavage stimulatory factor (CStF) and cleavage factors Im and IIm (CFIm and CFIIm) in humans, as well as cleavage factors IA and IB (CF IA and CF IB) in yeast (10–12). Other RNA-binding proteins regulate the length of the poly(A) tail and selection of alternative cleavage sites [known as alternative polyadenylation (APA)].

In this review, we first discuss the current mechanistic understanding of pre-mRNA 3′-end processing in eukaryotes, which has been greatly elaborated on by recent studies using structural biology and biochemical reconstitution of the 3 ^‘^-end-processing reaction. We next provide an overview of how pre-mRNA 3′-end processing is regulated and coordinated with other nuclear processes, particularly transcription and splicing. Finally, we highlight outstanding questions in the field of pre-mRNA 3′-end formation that remain to be addressed.

## 2 3′-End-Processing Machinery

### 2.1 CPSF/CPF Performs 3′-End Processing in Eukaryotes

Early studies aiming to understand the mechanistic basis of cleavage and polyadenylation of eukaryotic pre-mRNAs suggested that 3‘-end processing takes place within large protein complexes specifically assembled on the pre-mRNA substrate (13, 14). Fractionation of nuclear extracts enabled the identification of key 3′-end processing factors, and subsequent purification of endogenous protein complexes led to the determination of their subunit compositions (Table 1) (9, 15–19).

The subunits of the pre-mRNA 3′-end-processing machinery are highly similar across eukaryotes, highlighting their conserved function. Interestingly, the affinities between some components differ between yeast and humans, potentially allowing more regulation in multicellular eukaryotes (10). Human CPSF is composed of seven subunits (8, 17). The same subunits are found in yeast CPF, but it also contains eight additional proteins (9, 18). Both CPSF and CPF are composed of modules centered around the following enzymatic activities: endonuclease, poly(A) polymerase, and in the case of the yeast CPF complex, protein phosphatase activity (Figure 1d). Each module can be purified separately using recombinant proteins, but they are stably assembled into CPSF/CPF in cells.

#### 2.1.1 Polymerase module or mammalian polyadenylation specificity factor

The polymerase module, known as mammalian polyadenylation specificity factor (mPSF) in humans, is a structural scaffold for CPSF/CPF (8, 9). It recruits CPSF/CPF to pre-mRNAs and binds the poly(A) polymerase enzyme to mediate polyadenylation after endonucleolytic cleavage. mPSF, along with PAP, is sufficient for specific and efficient polyadenylation in vitro (20). Recent structural analyses using X-ray crystallography, cryo–electron microscopy (cryo-EM), and nuclear magnetic resonance spectroscopy have provided new insights into mPSF/polymerase module function (9, 21–26).

**Figure 2:**
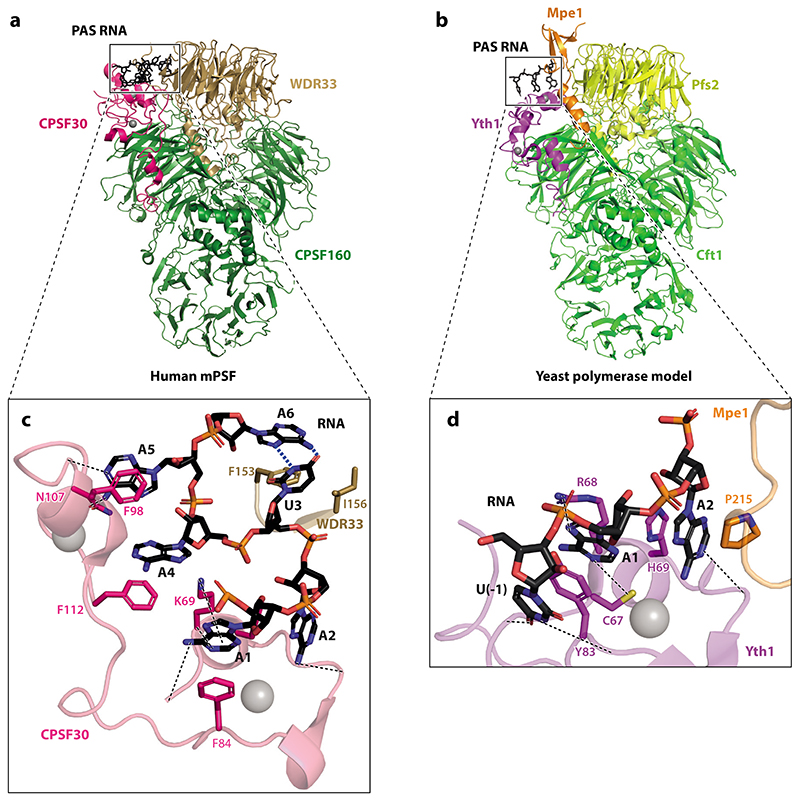
mPSF/polymerase module specifically recognizes the pre-mRNA. *(a,b)* Overall architecture of *(a)* human mPSF bound to an RNA containing a PAS sequence (PDB ID: 6DNH) and (*b*) the budding yeast polymerase module bound to PAS RNA and Mpe1 (PDB ID: 7ZGR) (21, 33). *(c,d)* Close-up view of the PAS RNA-binding sites of (*c*) human mPSF and *(d)* the yeast polymerase module. Some hydrogen bonds between the RNA and the protein subunits are indicated by dashed black lines. Hydrogen bonds that mediate Hoogsteen base pairing between U3 and A4 in the human complex are depicted in dashed blue lines. Some protein residues that make hydrophobic and stacking interactions with the RNA are also shown in stick representation. Zinc ions bound to ZnF domains of CPSF30/Yth1 are shown in gray. Abbreviations: CPSF, cleavage and polyadenylation specificity factor; mPSF, mammalian polyadenylation specificity factor; PAS, polyadenylation signal; PDB ID, Protein Data Bank identifier; ZnF, zinc finger.

mPSF contains four protein subunits: CPSF160, WDR33, hFip1, and CPSF30 (Cft1, Pfs2, Fip1, and Yth1 in the yeast polymerase module). Human PAP is not constitutively associated with the mPSF complex, but interestingly, Pap1 is a stable subunit in yeast. Structural analyses of both the human and yeast complexes have revealed details of their highly conserved architecture. CPSF160/Cft1 contains three β-propeller domains which are arranged in a trefoil configuration, with two of these domains forming a binding cavity for an N-terminal helical domain of WDR33/Pfs2 (Figure 2a,b). WDR33/Pfs2 also contains a β-propeller downstream of its helical domain. CPSF30/Yth1 interacts with the complex by contacting both CPSF160/Cft1 and WDR33/Pfs2 with two of its five zinc fingers (ZnFs).

The overall structures of mPSF and the polymerase module share a similar architecture but little sequence homology to the DNA damage–binding protein (DDB)1–DDB2 complex, which recognizes UV-damaged DNA, and the SF3b complex, which is part of the U2 small nuclear ribonucleoprotein (snRNP) involved in pre-mRNA splicing (9, 22). This suggests that these protein complexes structured around a β-propeller scaffold are used to facilitate binding to nucleic acids in eukaryotes.

#### 2.1.2 Recognition of the polyadenylation signal

Sites for 3′-end processing are marked by the hexameric polyadenylation signal (PAS) sequence (27). The PAS is located approximately 10–30 nucleotides upstream of the cleavage site and is often surrounded by auxiliary RNA motifs that bind cleavage factors (Figure 1c) (28). CPSF/CPF binds the PAS directly (20, 21, 23), and consequently, the sequence of the PAS determines the efficiency of 3′-end processing at a particular site. The consensus PAS sequence with the highest affinity for the 3′-end processing complex in most eukaryotes is AAUAAA (29, 30). However, only ~50% of PAS sites in humans contain the canonical AAUAAA motif (28, 31). Noncanonical PAS sequences are recognized less efficiently and may contribute to regulation of 3′-end processing.

The mechanism of PAS recognition by CPSF/CPF was first studied by UV cross-linking, which demonstrated that PAS RNA contacts the mPSF complex (20, 32). More recently, human mPSF bound to PAS-containing RNA was visualized using cryo-EM (21, 23). All six nucleotides of the AAUAAA sequence were visible, revealing that the hexamer interacts with ZnF2 and ZnF3 of CPSF30 and a surface on WDR33 (Figure 2c). Amino acid residues of CPSF30 form base-specific contacts with the RNA, facilitating the recognition of the PAS sequence. Additional specificity is provided by bases U3 and A6 of the PAS, which form a Hoogsteen base pair that inserts into a pocket of WDR33 and stabilizes the mPSF-bound conformation of the RNA.

A recent cryo-EM structure of the yeast polymerase module bound to a nuclease module subunit, Mpe1, showed how the 5′part of the PAS is recognized (Figure 2d) (33). The first two adenosines of the PAS were located at the same position as observed in the human complex, demonstrating that their recognition is highly conserved between yeast and human proteins. The in vitro affinity of the yeast polymerase module for RNA is approximately two orders of magnitude weaker than that of the human mPSF, suggesting that there are also some differences in recognition (12, 29). An additional nucleotide upstream of the PAS [U(–1)] was bound in a surface pocket of Yth1, raising the possibility that the nucleotides surrounding the PAS may also contribute to RNA binding and recognition by the polymerase module.

#### 2.1.3 Poly(A) polymerase

Polyadenylation of cleaved pre-mRNAs is catalyzed by PAP/Pap1 using a polymerization mechanism dependent on two catalytic magnesium ions (34). On its own, PAP displays only weak and distributive PAS-independent activity, likely because of its low affinity for RNA. mPSF stimulates PAP activity, likely by recruiting the enzyme to cleaved pre-mRNAs (20). Both PAP and Pap1 interact with mPSF/polymerase module via the hFip1/Fip1 subunit (35). A structure of residues 80–105 of yeast Fip1 bound to Pap1 has been determined, but additional residues also contribute to their interaction (24, 36). hFip1/Fip1 also interacts with CPSF30/Yth1 and thereby tethers Pap1 to the complex (24–26).

Fip1 is an intrinsically disordered protein in isolation, but some parts become structured upon binding to Yth1 and Pap1 (24–26, 37). The region of Fip1 connecting the Pap1 and Yth1 interaction sites has been shown to remain dynamic in the context of CPF (24). This may allow Pap1 to be flexibly tethered to the polymerase module and facilitate the addition of adenosines to a growing poly(A) tail. The dynamics of Fip1 may also explain why Fip1 and Pap1 were not resolved in cryo-EM maps of the polymerase module (9, 33). However, it remains to be determined whether Pap1 is flexibly tethered in the context of the fully assembled 3‘-end processing machinery on RNA.

Interestingly, native mass spectrometry of endogenous CPF identified a population of the complex bound to two, instead of just one, copies of Pap1 and Fip1 (9). Recent crystal structures of the human CPSF30–hFip1 complex revealed one copy of hFip1 binds to ZnF4 and a second copy binds to ZnF5 of CPSF30 (25, 26). The two copies of hFip1 enable tethering of two PAP molecules to a single mPSF complex. Further investigation is required to determine the functional significance of the nonuniform stoichiometry of PAP/Pap1 and hFip1/Fip1 in vivo.

#### 2.1.4 Nuclease module or mammalian cleavage factor

The nuclease module, known as mammalian cleavage factor (mCF) in humans, hosts the 3′endonuclease enzyme CPSF73/Ysh1; a pseudonuclease, CPSF100/Cft2; and a third nonenzymatic subunit (Figure 1d). In humans, this is a scaffold protein, symplekin (8). Its yeast ortholog, Pta1, also interacts with Ysh1 and Cft2 in a conserved manner, but Pta1 has been attributed to the phosphatase module of CPF based on native mass spectrometry data (9). This is consistent with the two modules being intimately associated. The yeast nuclease module contains Mpe1 as its third subunit. Interestingly, the human ortholog of Mpe1, RBBP6, is not a stable subunit of the CPSF complex (10).

**Figure 3:**
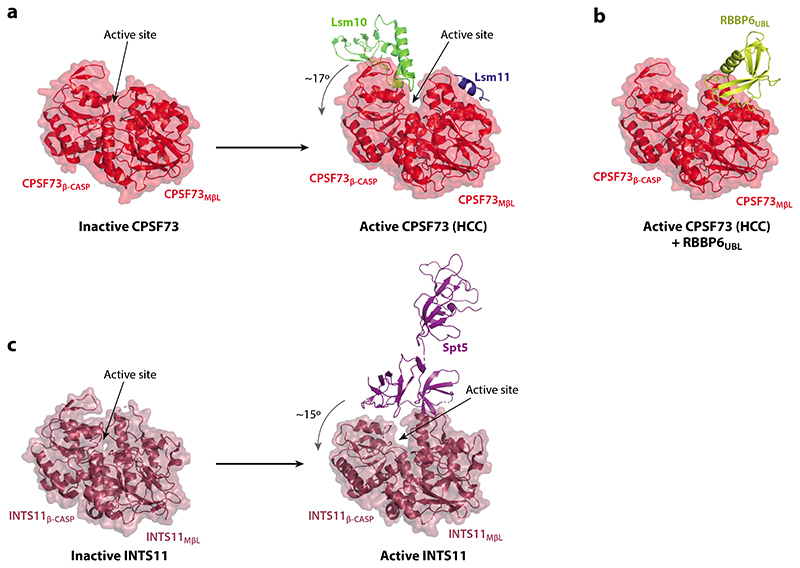
Activation of 3′processing endonucleases requires accessory factors. (*a*) Structural comparison between the inactive state of CPSF73 (PDB ID: 2I7T) (38) and activated CPSF73 (PDB ID: 6V4X) (45) bound to Lsm10 and Lsm11 within the HCC. Lsm10 acts as a wedge that induces a rotation of the MβL relative to the β-CASP domain, opening the active site of the endonuclease. (*b*) Structural model of activated CPSF73 bound to RBBP6, which is based on the experimental structure of the yeast dimeric complex (PDB ID: 6I1D) (12) and the HCC (PDB ID: 6V4X) (45). (*c*) Structural comparison between the inactive state of INTS11 (PDB ID: 7BFP) (43) within the Integrator cleavage module and activated INTS11 within Integrator bound to the paused Pol II complex (PDB ID: 7PKS) (62). Similarly to Lsm10, SPT5 promotes opening of the active site. Abbreviations: CPSF, cleavage and polyadenylation specificity factor; HCC, histone cleavage complex; MβL, metallo-β-lactamase domain; PDB ID, Protein Data Bank identifier; Pol II, RNA polymerase II.

CPSF73/Ysh1 and CPSF100/Cft2 contain metallo-β-lactamase and β-CASP domains (Figure 3a). The active site of CPSF73/Ysh1 is located in a cleft between these two domains where two catalytic zinc ions are coordinated by highly conserved amino acid side chains (38). The zinc-binding residues are less well conserved in CPSF100/Cft2, which has therefore been described as an inactive pseudonuclease (39). The endonuclease and pseudonuclease subunits form a constitutive dimer due to an interaction between their C-terminal domains (CTDs) that is stabilized by the binding of symplekin/Pta1 (8).

The trimeric complex containing CPSF73, CPSF100, and symplekin is also found within the histone pre-mRNA 3′-end-processing complex, where it is known as the histone cleavage complex (HCC) (40, 41). In addition, a very similar architecture, containing two subunits of the metallo-β-lactamase and β-CASP family and a scaffold protein, is found in the metazoan Integrator complex, which is involved in 3‘-end processing of noncoding RNAs and the resolution of Pol II paused at promoters (42–44). Thus, the architecture of mCF has been reused multiple times during evolution in protein complexes that cleave RNAs at their 3′ends.

mCF/nuclease module is connected to mPSF/polymerase module through the interaction of a highly conserved peptide of CPSF100/Cft2, called an mPSF-interacting motif (PIM), with a surface on CPSF160/Cft1 and WDR33/Pfs2 (8, 33). Electron microscopy analysis of recombinant complexes has revealed that the mCF/nuclease module does not occupy a fixed position relative to mPSF/polymerase module (8, 12). The inherent flexibility of CPSF/CPF may allow for processing of a large variety of eukaryotic pre-mRNAs, for example, with variable distances between the PAS and the cleavage site. It is possible that CPSF/CPF becomes more rigid in the presence of the RNA substrate and cleavage factors, forming a structured active complex.

CPSF73/Ysh1 adopts an inactive closed conformation in most of the structures that have been determined (Figure 3). The 3′endonuclease has only weak and nonspecific nuclease activity either in isolation or within the purified CPSF/CPF complex, consistent with it being in an inactive conformation (38). Recently, the histone pre-mRNA 3′-end-processing complex was visualized in its active state, revealing the mechanism of CPSF73 activation within HCC (Figure 3a) (45). However, due to the lack of conservation of nonenzymatic subunits between the histone pre-mRNA 3′-end-processing complex and CPSF, this structure does not explain how CPSF73 is activated in the context of CPSF. This remains a major outstanding question in the field.

#### 2.1.5 Protein phosphatases of 3′-end-processing complexes

The yeast phosphatase module incorporates two protein phosphatases, Ssu72 and Glc7, which target the highly conserved Y_1_S_2_P_3_T_4_S_5_P_6_S_7_ repeats on the CTD of Pol II (46). The phosphatase module also contains four additional nonenzymatic proteins: the symplekin ortholog Pta1, Swd2, Pti1, and Ref2 (9).

Incorporation of Ssu72 and Glc7 into a multi-subunit protein complex confers substrate specificity to the phosphatases. Ssu72 dephosphorylates the serine 5 and serine 7 residues of the CTD repeats during transcription elongation, while Glc7 promotes transcription termination, likely by dephosphorylating tyrosine 1, as well as a transcription elongation factor, Spt5 (47–50). Since CPF associates with actively transcribed protein-coding genes at promoters, CPF may contribute to the coordination of all stages of transcription (51).

Human cells express orthologs of both Ssu72 (SSU72) and Glc7 [protein phosphatase 1 (PP1)], but these proteins are not constitutively associated with CPSF. Nevertheless, SSU72 and PP1 both copurify with an RNA-bound 3′-end processing machinery, and they perform conserved functions in coordinating transcription with 3′-end processing (6, 46). Both human and yeast orthologs of Ssu72 interact with symplekin/Pta1 (52). Interestingly, in the absence of transcription, SSU72 inhibits 3′-end processing of histone pre-mRNAs but has no noticeable effect on cleavage activity by CPSF in vitro (10, 11, 45). In human cells, PP1 is part of a distinct protein complex that also contains WDR82, Tox4, and PNUTS subunits, the latter being a key regulator of the enzymatic activity of PP1 in transcription termination (Table 1) (53, 54).

A distinct protein complex called associated with Pta1 (APT) mediates transcription termination of noncoding small nuclear (sn)RNAs and small nucleolar (sno)RNAs in yeast (55). APT contains all the subunits of the phosphatase module but also contains a unique subunit, Syc1, which distinguishes APT from CPF. Syc1 is homologous to the CTD of Ysh1 and blocks the association of the nuclease and the polymerase modules with the APT complex. snRNAs and snoRNAs are neither cleaved nor polyadenylated, but the phosphatase activities of APT may regulate transcription of noncoding RNAs in the same way that the phosphatase module functions on protein-coding genes. snRNAs and snoRNAs are released from transcribing Pol II by the helicase activity of the Nrd1–Nab3–Sen1 complex (56).

In humans, 3′-end processing of snRNAs is executed by the Integrator complex, which does not share homology with APT (57). Integrator has been shown to resolve promoter-proximal pausing of Pol II at protein coding genes, preventing the transition of Pol II into productive elongation (58). Thus, Integrator regulates the production of mRNAs as well as snRNAs. It contains at least 14 subunits, including both endonuclease and protein phosphatase enzymatic activities (59). The Integrator–PP2A complex (INTAC) can dephosphorylate serine 2, serine 5, and serine 7 of the Pol II CTD in vitro, suggesting that a single phosphatase within INTAC could regulate both transcription elongation and transcription termination (59–61). The nuclease module of the Integrator complex has a highly similar architecture to mCF of CPSF, including the endonuclease INTS11, which contains metallo-β-lactamase and β-CASP domains, similar to CPSF73 (43, 59, 62).

The structure of the active RNA-bound Integrator complex associated with a paused Pol II elongation complex revealed that activation of the endonuclease subunit INTS11 requires the transcription elongation factor Spt5 (Figure 3b) (62). In contrast, the in vitro cleavage activity of CPSF/CPF does not require Pol II or its cofactors, suggesting that the CPSF endonuclease is activated by a different mechanism than the Integrator endonuclease.

### 2.2 Cleavage Factors Are Essential to CPSF/CPF Endonuclease Activation

The lack of specific and efficient endonuclease activity by the CPSF/CPF complex in isolation implied that the 3‘endonuclease requires an activation step. This could prevent nonspecific cleavage of RNAs in the nucleus and enable regulation of cleavage-site selection. Many potential regulatory factors have been identified using partially purified fractions of either mammalian or yeast nuclear extract (63–65). Subsequent in vitro reconstitution has defined exactly which protein factors are required for endonuclease activation (Table 1) (10–12). Almost all of the essential activator proteins appear to be highly conserved from yeast to humans.

#### 2.2.1 CStF, CFIIm/CF IA

CFIA is essential for the endonuclease activity of yeast CPF (12). It is composed of two copies each of the Rna14 and Rna15 subunits, which form a tetramer, and one copy each of Pcf11 and Clp1 (Figure 1d) (66, 67). In humans the orthologs of the CF IA subunits are present within two separate complexes: Human CStF complex contains two copies each of an ortholog of Rna14, CStF77; an ortholog of Rna15, CStF64; and a human-specific protein, CStF50 (68). Human Pcf11 and Clp1 are located within a heterodimeric CFIIm complex (69). Similar to their counterparts in yeast, both CStF and CFIIm are required for the endonuclease activity of human CPSF (10, 11).

CStF and CF IA bind G/U-rich elements located downstream of the cleavage site. RNA binding is mediated by the RRM domains of CStF64/Rna15, which have been shown to have a preference for G/U-rich sequences in vitro (70). Interestingly, CStF64 appears to be the only cleavage factor subunit that is stably associated with both endogenous human CPSF and a histone pre-mRNA 3′-end-processing complex purified directly from mouse and fly cells, most likely due to CstF64 interacting with symplekin (10, 41, 71). The significance of this finding remains unclear, since direct association with CStF64 is not required for endonuclease activity of either of these 3′-end-processing complexes in an in vitro setting (10, 11, 45). The half-a-tetratricopeptide repeat domains of CStF77 dimerize and interact with mPSF, contacting both the CPSF160 and WDR33 subunits (8). By binding both CPSF and specific sequence elements of the pre-mRNA substrate, CStF/CF IA may contribute to the specificity of cleavage-site selection and also position the RNA for endonucleolytic cleavage.

CFIIm may further increase the sequence specificity of the 3′processing machinery by binding to the G-rich element found on some pre-mRNAs further downstream of the G/U-rich motif (69). Pcf11 physically bridges CStF/Rna14–Rna15 and Clp1 and also interacts with the CTD of Pol II, likely helping to coordinate 3′-end processing with transcription (72, 73). Human Clp1 is an active polynucleotide kinase, while the yeast protein lacks the catalytic residues (74). The enzymatic activity of Clp1 is not essential for endonuclease activation, but some evidence indicates that ATP binding at its active site could be required (11).

Overall, CF IA/CStF and CFIIm are required to activate the 3′-end-processing endonuclease, but the mechanism of activation remains unknown.

#### 2.2.2 RBBP6/Mpe1

Yeast Mpe1 is part of the CPF nuclease module and is required for cleavage activity of the CPF complex (12). The human ortholog of Mpe1, RBBP6, is not a constitutive subunit of the human CPSF complex (10, 17). This meant that the role of RBBP6 in activating the endonuclease of CPSF was largely overlooked for many years. However, it also plays an essential role in pre-mRNA 3′-end processing: RBBP6 regulates alternative cleavage-site selection in cells, was detected in native postcleavage complexes bound to the 5′product of the cleaved pre-mRNA, and also interacts with CPSF in an RNA-dependent manner (6, 10, 75). Recently, RBBP6 was found to be essential for successful reconstitution of the human CPSF endonuclease activity with purified recombinant proteins (10, 11). Together with studies of the yeast cleavage reaction, this demonstrated that RBBP/Mpe1 is a highly conserved activator of CPSF/CPF endonuclease activity.

RBBP6 and Mpe1 contain a ubiquitin-like domain (UBL), a ZnF domain, and a RING finger domain. Human RBBP6 also carries a long, largely disordered C-terminal extension that is not required for activation of cleavage but may interact with transcription factors and splicing regulators (76–78). The UBL has been shown to interact with the metallo-β-lactamase domain of the endonuclease subunit in both yeast and humans (10–12). A crystal structure of the dimeric Mpe1 UBL–Ysh1 nuclease domain complex from yeast has been determined, showing that the UBL domain is located adjacent to the active site cleft of the endonuclease (Figure 3c) (12). It contributes a positively charged surface, which may direct the pre-mRNA substrate into the active site. Thus, the UBL domain of Mpe1/RBBP6 may directly contribute to the activity of the endonuclease enzyme in the context of the complete 3′-end-processing machinery.

A recent cryo-EM structure of the yeast polymerase module bound to Mpe1 and PAS RNA revealed that a different part of Mpe1 interacts with the Pfs2 subunit and, surprisingly, also contacts the PAS (Figure 2b,d) (33). Therefore, the region of Mpe1 that interacts with the polymerase module was referred to as the pre-mRNA sensing region (PSR). Mpe1 contacts RNA through a CH-π interaction between a proline residue and the A2 nucleotide of the PAS. This is a rather weak interaction, and therefore, Mpe1 affects neither the affinity of the polymerase module for RNA nor the sequence specificity of RNA binding. Instead, functional studies in vivo and in vitro showed that mutation of the proline resulted in defective pre-mRNA cleavage by CPF. Thus, Mpe1 may sense correct binding of PAS RNA to the polymerase module, coupling RNA recognition directly to the endonuclease through its UBL domain. The PSR of the human ortholog RBBP6 likely binds to mPSF in a similar manner (10).

Mpe1/RBBP6 is likely to cooperate with the previously described cleavage factor complexes in activating the endonuclease of CPF/CPSF, but little is known about how this could be achieved. Now that the 3‘endonuclease activity has been reconstituted in vitro with both yeast and human proteins and the factors required for this reaction have been identified, the next step is to determine the mechanistic and structural details of how CPSF, RBBP6, CStF, and CFIIm carry out the endonucleolytic cleavage of pre-mRNAs. Interestingly, Mpe1 is also predicted to bind to the poly(A) polymerase Pap1, and mutations in the PSR of Mpe1 lead to hyperpolyadenylation of a cleaved pre-mRNA substrate in vitro (33). Given its essential role in activating pre-mRNA cleavage, RBBP6/Mpe1 may coordinate the two 3′-end-processing reactions by the CPSF/CPF complex and may facilitate the potential conformational changes between the cleaving and polyadenylating states of CPSF/CPF.

#### 2.2.3 Nonessential cleavage factors

Additional cleavage factor proteins in both yeast and humans play regulatory roles in pre-mRNA 3‘-end processing. These factors tend to be specific to each species, suggesting that, while the basal cleavage and polyadenylation machinery is highly conserved, it may be regulated differently in yeast and humans (Table 1). Yeast CF IB consists of a single protein, Hrp1, that imparts specificity to CPF endonuclease activity by preventing secondary cleavage of the pre-mRNA substrate (12). CF IB binds to U-rich sequences upstream of the PAS, but further details of how it regulates cleavage are unclear.

In humans, the tetrameric CFIm complex contains two copies of the RNA-binding protein CFIm25 and two copies of either CFIm68 or CFIm59 (Figure 1d). CFIm favors the usage of poly(A) sites with upstream UGUA motifs by binding to these sequences and recruiting CPSF via an interaction between CFIm68 and hFip1 (79). Thus, CFIm contributes to alternative cleavage site selection and APA.

### 2.3 Nuclear Poly(A)-Binding Proteins Control Polyadenylation

The 3′-end processing machinery controls polyadenylation so that all poly(A) tails are synthesized to a relatively uniform length. The median length of a poly(A) tail of a mature mRNA upon nuclear export is species specific, varying between ~60–80 adenosines in budding yeast and ~250 adenosines in humans (80–82). Hyper- and hypopolyadenylated mRNAs are not exported from the nucleus and are degraded by the nuclear exosome (83, 84). Poly(A) tail length is likely regulated through accessory proteins and controlled processivity of PAP/Pap1 (Table 1).

Nuclear poly(A)-binding proteins, including Nab2 and PABPN1, contribute to normal control of poly(A) tail length. Once a poly(A) tail reaches ~60 adenosines in yeast and ~250 adenosines in humans, Nab2 and PABPN1, respectively, are thought to bind and inhibit processive polyadenylation (80, 85). In yeast, Pab1, which is less abundant in the nucleus than Nab2, can substitute for Nab2 and restrict the poly(A) tail length to ~90 adenosines if Nab2 is unavailable (80). In a current model, poly(A)-binding proteins promote PAP/Pap1 dissociation from RNA. However, a mechanistic understanding of poly(A) tail length control by poly(A)-binding proteins is still lacking.

Even in the absence of poly(A)-binding proteins, polyadenylation by yeast CPF can be restricted to ~ 100–200 nucleotides in vitro and in cells (80). The mechanistic basis of such intrinsic length control remains unclear, but physiologically, it is thought to act as a fail-safe mechanism in yeast under stress conditions when Nab2 and Pab1 are depleted. A similar restriction of poly(A) tail length is observed when the concentration of PABPN1 in the nucleus is decreased in mammalian cells, suggesting that CPSF also exhibits intrinsic length control of polyadenylation. In the absence of PABPN1, CPSF is less processive, and its rate of adenosine addition decreases as the distance between the PAS and the 3′end of the poly(A) tail increases (85). Thus, the mechanisms of intrinsic poly(A) tail length control may be conserved between yeast and humans.

### 2.4 Histone Pre-mRNA Processing and Insights into CPSF73 Activation

Not all protein-coding mRNAs in eukaryotes are polyadenylated. 3′-end processing of metazoan replication-dependent histone pre-mRNAs involves endonucleolytic cleavage downstream of a conserved stem loop structure bound by the stem loop–binding protein (SLBP). SLBP is upregulated prior to the onset of the S phase, when genes encoding replication-dependent histones are transcribed (86). SLBP promotes 3′-end processing of histone pre-mRNAs, as well as their export, translation, and stability (87, 88). This allows coordinated production of replication-dependent histone mRNAs specifically during the S phase of the cell cycle when histones are required for chromatin assembly during DNA replication.

3′-end processing of replication-dependent histone pre-mRNAs is executed by a specialized ribonucleoprotein machinery. The histone pre-mRNA 3′-end-processing complex contains a seven subunit Sm ring bound to U7 snRNA; subunits Lsm10, Lsm11, FLASH, and SLBP; and the HCC subcomplex that is composed of the same subunits as mCF, including endonuclease CPSF73 (41, 89). CStF64 has also been shown to be associated with the histone-processing complex, but the functional consequences of this interaction remain unknown. Despite sharing the endonuclease subunit, the histone pre-mRNA 3′-end-processing complex and CPSF differ in their mechanisms of both RNA recognition and endonuclease activation (45). For example, unlike protein-mediated PAS recognition by CPSF, the U7 snRNA within the histone complex recognizes a conserved histone downstream element (HDE) in the pre-mRNA using canonical base pair interactions. The HDE duplex is ~15 nucleotides long, which, along with the recognition of the stem loop structure by SLBP, ensures highly specific recognition of histone pre-mRNAs.

A recent cryo-EM structure of the active histone pre-mRNA 3′-end-processing machinery revealed large-scale conformational rearrangements within the HCC complex. The activation mechanism involves the opening of the CPSF73 active site due to the metallo-β-lactamase and β-CASP domains pivoting away from each other (Figure 3a) (45). This allows substrate RNA to access the active site, while the positions of the key catalytic residues remain unchanged. The N-terminal domain of symplekin is essential for this activation but is dispensable for CPSF cleavage activity in vitro (10). Lsm11, a subunit unique to the histone pre-mRNA processing complex, interacts with the metallo-β-lactamase domain of CPSF73 to promote activation. The same surface is likely bound by RBBP6 in the CPSF complex (Figure 3c) (10–12). Also, Lsm10 forms contacts with the β-CASP domain of CPSF73. This is reminiscent of the transcription elongation factor SPT5 interaction with INTS11 that may trigger the opening of INTS11 and the activation of the Integrator endonuclease (Figure 3d) (62, 90). By analogy, CPSF73 within the canonical 3‘-end-processing machinery is likely activated by combined binding of the RBBP6 UBL to the metallo-β-lactamase domain and binding of another interactor to the β-CASP domain. An active state structure of CPSF bound to its substrate and auxiliary activators is essential to determine how the active site of CPSF73 is pried open within the canonical 3′-end-processing machinery.

## 3 Regulation of 3′-End Processing

**Figure 4:**
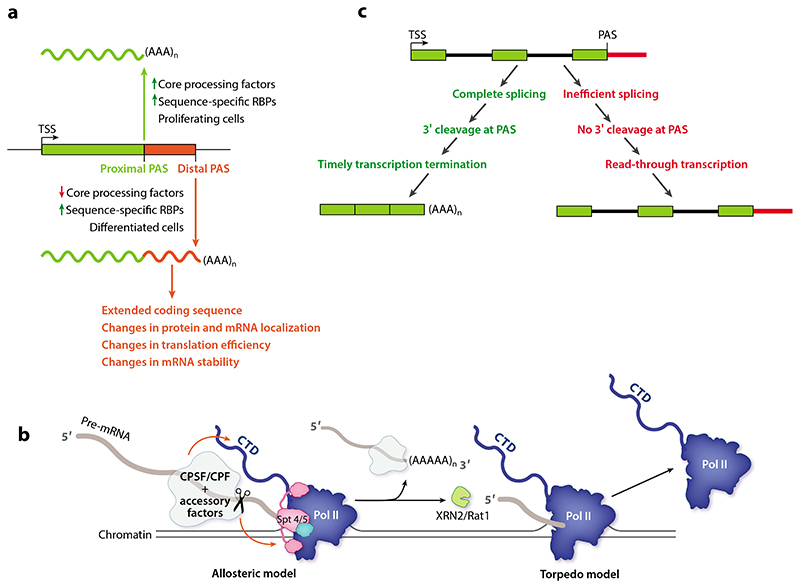
Eukaryotic pre-mRNA 3‘-end processing is tightly regulated and coordinated with splicing and transcription termination. (*a*) Schematic representation of APA showing the factors that influence the choice of cleavage site. (*b*) Schematic representation of transcription termination. Red arrows depict phosphatase activity of CPSF/CPF acting on the CTD of Pol II and on transcription elongation factor SPT5. (*c*) Schematic representation of coupling between splicing, 3′-end processing, and transcription termination. Panel *b* adapted from Reference 93. Abbreviations: APA, alternative polyadenylation; CPF, cleavage and polyadenylation factor; CPSF, cleavage and polyadenylation specificity factor; CTD, C-terminal domain; PAS, polyadenylation signal; Pol II, RNA polymerase II; RBP, RNA-binding protein; TSS, transcription start site.

Approximately 70% of protein-coding genes in both budding yeast and metazoans produce several mRNA isoforms that differ in the sequence of their 3′ends (91). Although this has been traditionally termed APA, the specific selection of alternative cleavage sites by the CPSF/CPF complex is at the heart of generating alternative 3′ends of the same mRNA (Figure 4a). APA has a regulatory capacity to tune when, where, how much, and which protein is translated from each mRNA and is therefore highly regulated based on cell type, developmental stage, and cellular conditions (2).

APA can change the identity of the protein product if cleavage occurs before the stop codon. This can, for example, produce either a protein lacking its C-terminal regions, often affecting its function, or a truncated, nonfunctional polypeptide. In contrast, APA after the stop codon does not alter the amino acid sequence of the translated polypeptide but instead changes the length and sequence of the 3′UTR of the mRNA with potential consequences for its stability and localization. For instance, alternative mRNA isoforms with altered 3′UTR lengths often display different decay rates in the cytoplasm.

3′UTRs commonly include complementary binding sites for microRNAs, as well as sequence motifs (for example, AU-rich elements) recognized by RNA-binding proteins that target mRNAs for deadenylation and subsequent decay (92). Therefore, isoforms with shorter 3′UTRs are often more stable than their counterparts carrying longer 3′UTRs. In addition, 3′UTRs may contain elements targeting the transcript, and thereby its protein product, to a particular subcellular location. Many neuronal transcripts (for example, *Importin β1* and *Bdnf)* have isoforms containing longer 3′UTRs with preferential localization to neuronal projections compared to the cell body (94). Recently, it has been shown that 3′UTRs may affect the localization of the translated protein independent of mRNA localization. The 3′UTR of the long isoform of *CD47* mRNA has been demonstrated to act as a binding platform for proteins that cotranslationally assemble with its protein product and target the CD47 protein to the plasma membrane (95).

APA has been covered in several excellent reviews, and we do not discuss it in detail here (2, 3). In brief, in a kinetic model of PAS recognition, changes in the cellular concentrations of the 3′-end-processing machinery can alter the cleavage site. An increased abundance of CPSF/CPF or accessory factors may change the RNA-binding landscape to promote the use of proximal PAS sites that emerge early from Pol II but are often suboptimal. In contrast, lower cellular concentrations of the 3′-end-processing machinery enforce the use of distal, canonical PAS sites. However, it was recently shown that sequential cleavage can occur on some transcripts: Some pre-mRNAs that are cleaved using a distal PAS first are retained in the nucleus until they are subsequently processed a second time posttranscriptionally using a proximal PAS (96).

The kinetic model would likely have a global effect on cleavage sites. For example, mRNAs in proliferating cells generally have shorter 3′UTRs, while in differentiated cells, distal PAS sequences tend to be used more often (97). In agreement with this, cell types that tend to express higher levels of proteins that are part of the core 3′-end-processing machinery show a preference for proximal PAS sequences (98).

Regulation of APA by varying expression levels of core 3′-end-processing factors appears to be widespread, and many specific examples have been documented. For example, during B cell differentiation, elevated levels of CStF64 promote a switch from using the distal PAS to the proximal PAS, which leads to the production of IgM antibody missing its transmembrane domain, allowing the antibody to be secreted (99). Interestingly, many 3^′^-processing factors (including Pcf11, CStF77, and RBBP6) control their own expression by APA, resulting in a negative autoregulatory feedback loop (75, 100, 101).

Although variations in the cellular concentrations of core 3‘-end-processing factors have a global effect, the sensitivity of individual transcripts depends on the sequence motifs they contain. For instance, changes in CStF expression may have a greater effect on APA of polyadenylation sites containing GU-rich elements normally targeted by CStF. This could explain some transcript-specific effects of experimental depletion or overexpression of core factors.

A second mechanism for APA involves accessory binding proteins that either promote or repress certain cleavage sites. For example, sequence-specific RNA-binding proteins such as NOVA2, ELAV, FUS, and SR proteins regulate APA of their target RNAs (2). How these proteins affect the core 3‘-end-processing machinery to regulate APA is largely unknown. The best studied sequence-specific APA regulator is the CFIm complex. The CFIm25 subunit recognizes UGUA motifs that are enriched upstream of distal polyadenylation sites. CFIm promotes production of long 3′UTR isoforms by recruiting CPSF via an interaction between CFIm68 and the hFip1 subunit of CPSF (79). An alternative CFIm subunit, CFIm59, can partially rescue CFIm68 depletion but appears to be a weaker activator of 3′-end processing, suggesting that the relative expression levels of CFIm68 and CFIm59 may fine-tune APA regulation.

The simplicity of the RNA sequence motifs required for the recruitment of the 3′-end-processing machinery means that CPSF and cleavage factors may assemble on many sites along the nascent pre-mRNA other than the true PAS. To ensure that full-length transcripts are produced, cleavage at such cryptic 3′-end-processing sites in the gene body must be prevented. Usage of intronic cleavage sites is inhibited by U1 snRNP bound to 5′splice sites (102, 103). In the process called telescripting, U1 snRNP mediates the exchange of CFIm68 for CFIm59 in 3′-end-processing complexes, rendering them inactive (102). Interestingly, intronic polyadenylation as a gene regulatory mechanism is rather prevalent and has been observed globally under heat-shock conditions, as well as at specific genes (104).

### 3.1 Regulation of 3′-End Processing in Disease

3′-end processing is deregulated in disease. Mutations in genes encoding components of the 3′-end-processing machinery are found in cancer, neurological disease, and developmental disorders, and their expression is also often misregulated (105). Point mutations in the sequences that specify the cleavage site can also lead to disease. For example, a single point mutation in the PAS of the globin gene alters the cleavage site, leading to destabilization of the globin transcript and a disease known as thalassemia (106).

#### 3.1.1 CPSF73 as a therapeutic target

Recent years have seen a growing interest in targeting 3′-end processing pharmacologically, in particular by inhibiting the endonuclease activity of the CPSF complex (107). Compounds with anticancer, antiinflammatory, and antiprotozoan properties have been shown to bind in the active site of CPSF73 and compete with substrate RNA, thereby inhibiting endonucleolytic cleavage (108–112). For instance, benzoxaborole compounds, such as AN3661, specifically inhibit protozoan orthologs of CPSF73 and have the potential to treat malaria and toxoplasmosis (111, 112). Despite the high degree of sequence conservation among CPSF73 orthologs, small differences in the conformation of their active sites enable AN3661 to selectively inhibit protozoan enzymes without causing severe toxicity to the human host (111).

Another compound that targets CPSF73, JTE-607, prevents inflammation and also inhibits growth of Ewing′s sarcoma, acute myeloid leukemia, and pancreatic ductal adenocarcinoma cell lines (109, 113). JTE-607 induces global read-through transcription and R-loop formation in cancer cells (109). Many types of cancers overexpress CPSF73 (113, 114). The elevated transcriptional activity of cancer cells may make their survival more dependent on pre-mRNA processing than that of nontransformed tissues, providing a therapeutic window where cancer cells are more susceptible to inhibition of CPSF73. Proximal polyadenylation sites tend to be less optimal, and therefore, they may be more sensitive to inhibition of CPSF73. Thus, JTE-607 may also prevent growth of cancer cells by restoring the usage of more optimal distal polyadenylation sites seen in healthy tissues, but this has not yet been investigated. In contrast, in other cancers, such as renal clear cell carcinoma, transcriptional read-through is widespread, explaining why inhibition of the 3′endonuclease might not be effective against all cancer types (115).

Overall, CPSF73 has emerged as a druggable node in both transformed human cells and eukaryotic parasites. Structural studies have already illuminated how some clinically important compounds inhibit isolated CPSF73 (109, 111). Use of an in vitro reconstituted 3′-end-processing reaction with purified proteins for compound screening may allow the identification of new drugs that target 3′-end processing. Such a high-throughput system has been recently established for the histone pre-mRNA 3‘-end-processing reaction (116), and the recently reconstituted endonuclease activity by the CPSF complex may follow suit. It will be exciting to see if any novel CPSF73 inhibitors enter clinical use in the near future.

#### 3.1.2 The 3′-end-processing machinery is targeted by viruses

3′-end processing of pre-mRNAs is a vital step in gene expression, and thus, several viruses, including influenza A and herpes simplex virus 1 (HSV-1), have evolved mechanisms to interfere with 3′-end processing of host transcripts. Infection with influenza or HSV-1 causes transcriptome-wide inhibition of 3′-end processing leading to defects in transcription termination and pervasive read-through transcription (117, 118). Such read-through transcription results in global downregulation of host gene expression, allowing the virus to take over the cellular machinery for its own amplification. Single proteins within each virus, nonstructural protein 1 (NS1) in influenza A and ICP27 in HSV-1, are thought to be the primary inhibitors of 3′-end processing, because ectopic overexpression of these proteins is sufficient to replicate most of the 3′-end-processing defects observed in the respective viral infections (117, 119).

Both NS1 and ICP27 directly interact with the CPSF complex, but their inhibitory mechanisms do not seem to be conserved. NS1 binds ZnF2 and ZnF3 of CPSF30 and may compete with PAS recognition, preventing CPSF recruitment to pre-mRNAs (120). ICP27 has been proposed to interact with hFip1 and CPSF73 and prevent the assembly of productive 3′-end-processing machinery (117). On the other hand, ICP27 stimulates cleavage and polyadenylation of viral transcripts, and certain sequence motifs found upstream of the PAS of viral pre-mRNAs have been implicated in enabling ICP27 to act as an activator of viral 3′-end processing. Further studies are required to explain how ICP27 discriminates viral transcripts from host pre-mRNAs and either stimulates or inhibits cleavage and polyadenylation. In-depth understanding of how viruses affect 3′-end processing in their metazoan host may also aid in the development of new therapeutics.

## 4 Impact of 3′-End Processing on Gene Expression

### 4.1 Coordination with Transcription Termination

3‘-end processing of nascent RNAs is closely linked to transcription termination, since defects in 3^′^-end cleavage caused by a variety of perturbations all result in transcription read-through (121). The dependence of transcription termination on PAS recognition was postulated more than 30 years ago, and we are now beginning to obtain insight into the molecular mechanism of the coordination of these two events on protein-coding genes (Figure 4b) (122).

A prevailing model of transcription termination in eukaryotes posits that Pol II changes into a termination-competent complex after the PAS has been transcribed (the allosteric model). This allosteric change likely causes Pol II to slow or pause. Subsequent cleavage of the 5′-capped pre-mRNA by CPSF/CPF and its release from the transcribing Pol II leaves an unprotected 5′phosphate on the nascent RNA, which is then targeted for degradation by a processive 5′-to-3′torpedo exonuclease, XRN2. The reduced rate of Pol II progression along the DNA template allows XRN2 to catch up with the polymerase and displace it from chromatin, terminating transcription (the torpedo model). The 3′-end-processing machinery is critical both for the deceleration of the transcribing polymerase and for providing access to XRN2 (123). The PNUTS-PP1 complexhas been demonstrated to reduce the rate of Pol II progression by dephosphorylating the C-terminal region of the transcription elongation factor SPT5 (53). In yeast, CPF-mediated dephosphorylation of tyrosine-1 of the CTD of Pol II at the 3′end of protein-coding genes promotes recruitment of Pcf11 and Rtt103, which interact with the yeast homolog of the XRN2 exonuclease, Rat1 (47). In addition, recent work shows that dephosphorylation promotes Pol II dimerization (93). This dimer is compatible with basal transcription but not with the binding of transcription elongation factors. Therefore, Pol II dimerization may be the allosteric change that promotes transcription termination. Overall, the phosphatase activity of PNUTS–PP1/Ref2–Glc7 may contribute to transcription termination by acting on several different substrates.

A major outstanding question is how the CPSF/CPF phosphatase activities are triggered after transcription of the PAS. The PNUTS–PP1 complex is strongly enriched on the chromatin regions around annotated 3′cleavage sites, suggesting that specific recruitment of PNUTS–PP1 by the fully assembled 3′-end-processing complex may lead to dephosphorylation of Spt5 around the site of 3′-end cleavage (53). However, cleavage activity by CPSF/CPF is not strictly required for PAS-dependent transcription termination (124, 125).

The cleavage and polyadenylation machinery may also have roles in transcription termination beyond mediating Pol II deceleration and XRN2 accessibility (Figure 4b). Coimmunoprecipitation studies have detected physical association between Pol II and CPSF subunits, and recent work shows that Pol II and CPF interact directly in vitro (93, 126). Pcf11 also interacts with the CTD of Pol II (73). Now that both 3′-end processing and transcription have been reconstituted with purified components in a test tube, it will be possible to test hypotheses regarding the coordination of 3′-end processing and transcription termination in a minimal in vitro system.

### 4.2 Delayed Transcription Termination When 3′-End Processing Is Misregulated

A decrease in 3′-end processing efficiency by various perturbations, such as heat and osmotic shock, as well as viral infections, leads to transcriptional read-through due to concomitant defects in transcription termination. Read-through transcription can produce a unique class of noncoding RNAs called downstream of gene (DoG) transcripts (127). These chimeric transcripts consist of the sequence of the gene where transcription was initiated plus downstream genomic regions, extending >5 kb past the annotated 3′-end-processing site. The genes that produce DoGs are highly correlated among various stress conditions and include genes with weak PAS sequences and certain chromatin marks. Closely spaced genes also appear to be more prone to read-through transcription.

It remains unclear how general stresses such as hyperosmosis or heat shock impair or alter the function of the 3′-end-processing machinery to induce DoGs. Experimental depletion of both canonical factors and Integrator subunits has been shown to produce DoGs from proteincoding genes. Interestingly, hyperosmosis reduces the association between transcribing Pol II and the Integrator complex, while its interactions with canonical 3′-end-processing factors remain unaffected (128). Since the activity of the Integrator complex is primarily restricted to promoter-proximal regions, the upregulation of DoGs upon its depletion seems puzzling (129). It is possible that transcriptional read-through under stress conditions could also be caused by aberrant Pol II elongation complexes that would have been terminated by Integrator early in transcription but instead escaped promoter-proximal pausing (58).

The functional consequence of DoG transcription also remains unclear. Since DoGs tend to remain associated with chromatin, it has been hypothesized that DoG production may affect the three-dimensional organization of chromatin, and DoGs themselves may help reinforce the structure of the nucleus by interacting with nuclear scaffold proteins (130). Future discoveries regarding the functional role of read-through transcripts may add to the growing list of regulatory effects that 3′-end processing has on gene expression.

### 4.3 Transcriptional Interference as a Consequence of Inefficient 3′-End Processing

If read-through transcription extends into a neighboring gene, the rate of transcription at this next gene might be reduced due to transcriptional interference (33, 131). Mechanistically, transcribing Pol II may either displace transcription initiation factors bound to a downstream promoter, blocking transcription initiation (if both genes are transcribed in the same direction), or collide with a Pol II transcribing in the opposite direction (if the two genes are transcribed from different DNA template strands). Many noncoding transcription events are thought to regulate gene expression by transcriptional interference.

Genes are more closely spaced in the yeast genome than in the human genome (132), where transcription may continue for several kilobases without running into a downstream gene. This renders the yeast genome more susceptible to transcriptional interference and may demand a more efficient mechanism of transcription termination. Indeed, yeast CPF appears to be a more efficient endonuclease than human CPSF in vitro (10–12). Also, many proteins critical for 3′-end cleavage (Mpe1) and transcription termination (phosphatase module subunits, including Ref2-Glc7) are constitutively bound to yeast CPF but not to human CPSF. Instead, they are recruited to the 3′ends of protein-coding genes by the human machinery. Thus, an increased efficiency of the yeast 3′-end-processing complex may minimize transcriptional interference in the highly compact yeast genome.

### 4.4 Coordination with Splicing

Recent advances in nascent RNA sequencing methods have revealed unexpected coordination between cotranscriptional splicing and 3′-end processing of eukaryotic pre-mRNAs. Long-read sequencing of chromatin-associated RNA has enabled the elucidation of full-length pre-mRNA processing intermediates, allowing unambiguous determination of their splicing status and the position of Pol II along the gene at the time of sample preparation (133–136). These studies have uncovered the all-or-none nature of pre-mRNA processing: Fully spliced pre-mRNAs are successfully cleaved at their 3‘ends and do not display read-through transcription, while transcripts that retain introns tend to be cleaved inefficiently, as indicated by transcription continuing far beyond their annotated PAS (Figure 4c) (133). This all-or-none coupling suggests extensive cross talk between the splicing and the 3′-end-processing machineries. Since cotranscriptional splicing precedes 3′-end processing, the removal of introns has been suggested to improve the efficiency of 3′-end processing. The influence of splicing on 3′-end cleavage has been demonstrated by studying the effects of a mutation found in some patients with β-thalassemia. A point mutation located in the intron of the β-globin gene creates a cryptic 3′splice site that is used more efficiently than the canonical 3′splice site. The increased splicing efficiency of the intron also leads to improved usage of the annotated PAS and reduced transcriptional read-through of the β-globin gene (133).

How splicing stimulates 3′-end processing remains unknown. It has been suggested that splicing alleviates constitutive inhibition of 3′-end cleavage. A candidate inhibitor of 3′-end cleavage is the U1 snRNP that is bound to the 5′splice site of introns. U1 snRNP interacts with transcribing Pol II and also prevents intronic polyadenylation at cryptic PAS sites by inhibiting the assembly of an active 3′-end-processing complex. The removal of U1 snRNP after successful splicing of the 3′-terminal intron may then allow efficient 3′-end cleavage (102, 137). Other proteins that may inhibit 3′-end processing until splicing is complete include U2AF, PTB, and hnRNPC. It is also possible that the proteins that are deposited on pre-mRNAs after successful excision of introns, such as the exon junction complex and SR proteins, activate 3′-end processing. In addition, the coupling between splicing and 3′-end processing could be indirect, for example, mediated by the kinetics of processing events, RNA secondary structures, or the local chromatin landscape.

Read-through transcripts that remain unspliced are generated at low levels even in unperturbed cells in the absence of external stress (135). Such unprocessed RNAs are typically retained in the nucleus and degraded by the nuclear exosome. Thus, inefficient pre-mRNA processing effectively downregulates mRNA levels independently of transcription initiation and may act as a regulatory mechanism of gene expression.

## 5 Conclusions and Perspectives

**Figure 5:**
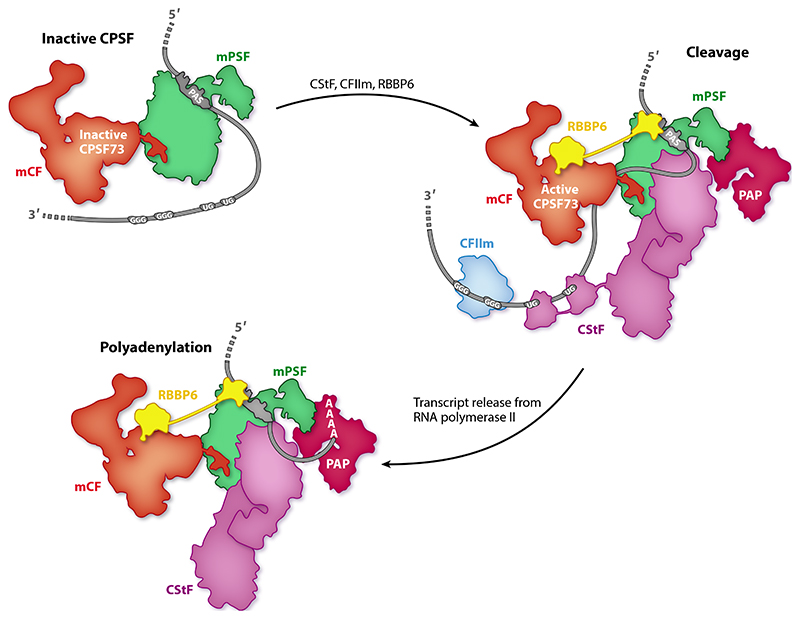
Models of the architecture of the eukaryotic 3‘-end processing machinery before activation, in its active cleavage state, and during polyadenylation. The order in which individual protein factors assemble is unknown. Abbreviations: CF, cleavage factor; CPSF, cleavage and polyadenylation specificity factor; CStF, cleavage stimulatory factor; mCF, mammalian cleavage factor; mPSF, mammalian polyadenylation specificity factor; PAP, poly(A) polymerase; PAS, polyadenylation signal.

3‘-end processing of pre-mRNAs plays a critical role in gene expression in eukaryotes. The relatively simple cleavage and polyadenylation reactions require at least a dozen polypeptides. This rather elaborate machinery ensures the fidelity and specificity of 3‘-end processing. In vitro reconstitution and structural studies using a variety of techniques have provided a glimpse into the architecture and mechanism of the 3′-end-processing machinery in both yeast and humans (Figure 5). However, the structures of the complete active machinery at various stages of 3′-end processing are still awaiting elucidation. Beyond 3′-end processing, mounting functional evidence has revealed that cleavage and polyadenylation are tightly coupled to transcription and splicing. Future mechanistic studies will hopefully reveal the mechanistic basis of how 3′-end processing is coordinated with the other steps of pre-mRNA biogenesis.

## Figures and Tables

**Table 1:** Canonical pre-mRNA 3′-end-processing factors in humans and budding yeast, their functions, and the multi-subunit protein complexes to which they belong. Alternative names are in brackets

Human complex		Human protein	Function	Yeast protein	Yeast complex		
CPSF	mPSF	CPSF160 (CPSF1)	Scaffold	Cft1	Poly(A) polymerase module	Core CPF	CPF
		WDR33	Scaffold, RNA binding	Pfs2			
		CPSF30 (CPSF4)	RNA binding	Yth1			
		hFip1	PAP/Pap1 recruitment	Fip1			
	N/A	PAP	Poly(A) polymerase	Pap1			
		RBBP6	Endonuclease activation	Mpe1	Nuclease module		
	mCF	CPSF100 (CPSF2)	Pseudonuclease	Cft2			
		CPSF73 (CPSF3)	Endonuclease	Ysh1			
		Symplekin	Scaffold	Pta1	Phosphatase module	APT	
N/A	N/A	SSU72	Protein phosphatase	Ssu72			
	Phosphatase complex	WDR82	Transcription termination	Swd2			
		PP1	Protein phosphatase	Glc7			
		PNUTS	Scaffold, PP1/Glc7 activator	Ref2			
		N/A	Scaffold	Pti1			
		Tox4	DNA binding	N/A	N/A		N/A
	N/A	N/A	Ysh1 antagonizing	Syc1			
	CStF	CStF50 (CSTF1)	Complex stabilizing	N/A		N/A	
		CStF64 (CSTF2)	RNA binding	Rna15	CF IA		
		CStF77 (CSTF3)	CPSF160/Cft1 binding	Rna14			
	CFIIm	Pcf11	Pol II CTD binding	Pcf11			
		Clp1	Polynucleotide kinase	Clp1			
		N/A	Cleavage fidelity	Hrp1	CF IB		
	CFIm	CFIm25 (CPSF5)	RNA binding	N/A	N/A		
		CFIm68 (CPSF6)	hFip1 binding	N/A			
		CFIm59 (CPSF7)	hFip1 binding	N/A			
	N/A	PABPN1	Poly(A) tail binding	N/A			
		N/A	Poly(A) tail binding	Pab1			
		ZC3H14	Poly(A) tail binding	Nab2			

Abbreviations: APT, associated with Pta1; CF, cleavage factor; CPF, cleavage and polyadenylation factor; CPSF, cleavage and polyadenylation specificity factor; CStF, cleavage stimulatory factor; CTD, C-terminal domain; mCF, mammalian cleavage factor; mPSF, mammalian polyadenylation specificity factor; N/A, not applicable; Pol II, RNA polymerase II; poly(A), polyadenosine; pre-mRNA, precursor messenger RNA.
